# Phase Responses of Oscillating Components in a Signaling Pathway

**DOI:** 10.3389/fphys.2013.00068

**Published:** 2013-04-05

**Authors:** Masaki Nomura, Mariko Okada-Hatakeyama

**Affiliations:** ^1^Laboratory for Cellular Systems Modeling, RIKEN Research Center for Allergy and ImmunologyYokohama, Japan

**Keywords:** oscillation, phase response analysis, synchrony, signal transduction, MAPK

## Abstract

Signal transduction pathways control various events in mammalian cells such as growth, proliferation, differentiation, apoptosis, or migration in response to environmental stimuli. Because of their importance, the activity of signaling pathways is controlled by multiple modes of positive and negative feedback regulation. Although negative feedback regulation primarily functions to stabilize a system, it also becomes a source of emerging oscillations. For example, the oscillatory behavior of mitogen-activated protein kinase (MAPK) activity has been theoretically proposed earlier and experimentally verified recently. However, the physiological function of such oscillatory behavior in biological systems remains unclear. To understand the functional aspects of this behavior, one should analyze the oscillation dynamics from a mathematical point of view. In this study, we applied the phase reduction method to two simple, structurally similar phosphorylation-dephosphorylation cycle models with negative feedback loops (Models A and B) and a MAPK cascade model, whose dynamics all show oscillation. We found that all three models we tested have a Type II phase response. In addition, we found that when a pair of each models A and B coupled through a weak diffusion interaction, they could synchronize with a zero phase difference. A pair of MAPK cascade models also showed synchronous oscillation, however, when a time delay was introduced into the coupling, it showed an asynchronous response. These results imply that structurally similar or even identical biological oscillators can produce differentiated dynamics in response to external perturbations when the cellular environment is altered. Synchronous or asynchronous oscillation may add strength to or dampen the efficiency of signal propagation, depending on subcellular distances and cell density. Phase response analysis allows prediction of dynamics changes in oscillations in multiple cellular environments.

## 1 Introduction

Oscillatory dynamics are widely distributed in nature (Strogatz, 2003). In biological systems, circadian rhythm, heart rhythm, locomotion, and electrical activity of the brain are well-known oscillation generators (Winfree, 2001; Khalsa et al., 2003). Although oscillatory behavior is a product of negative feedback regulation, the question of how the oscillatory information is processed in biological systems is still unresolved. Mammalian cells respond to extra-cellular signals and transfer this information to the nucleus to express/repress genes necessary for adaptation to a new environment or differentiation state. Signal transduction pathways play important roles to control expression of the correct genes and with the precise timing to satisfy cellular needs. Therefore, signaling pathways are spatio-temporally controlled by many positive and negative feedback loops through transcriptional and post-transcriptional modification. As a result, several types of oscillatory behaviors in the components of signaling pathways can be observed when negative feedback regulation is introduced into a system. For example, Shankaran et al. have shown persistent periodic shuffling of fluorescent-labeled extra-cellular signal-regulated kinase (ERK, a subset of the family of mitogen-activated protein kinases, MAPK) between cytosol and nucleus in epidermal growth factor (EGF) stimulated cells at the single cell level. Intriguingly, these periodic cycles among neighboring cells were asynchronous (Shankaran et al., 2009). ERK is one of the deterministic kinases that control transcription when translocated into the nucleus, therefore this nuclear shuffling process is highly regulated. The work of Shankaran et al. was the first experimental demonstration of the oscillatory behavior of ERK, although this was predicted earlier on theoretical grounds (Kholodenko, 2006). Given this asynchronous oscillation, one would think that it would be difficult to identify ERK dynamics in a population of cells, where the signal would be averaged. In addition, since periodic activation of ERK has been difficult to demonstrate experimentally, the cellular conditions leading to oscillatory ERK activation are likely quite narrow and restricted. In general, when oscillators interact with each other through a strong coupling, they tend to synchronize. Therefore, the asynchronous oscillation observed by Shankaran et al. suggests that the coupling strength of ERK in neighboring cells is weak, at least under the experimental conditions used in these studies. The question then arises, what kind of conditions allows a pair of cells to achieve asynchronous oscillation? Is the weak coupling enough to cause asynchronous oscillation? We have investigated these questions by applying the phase reduction method to two models of phosphorylation-dephosphorylation cycles and in a MAPK cascade, all of which exhibit negative feedback regulation.

The mechanism that causes the emergence of oscillations has been energetically studied (for example, Guckenheimer and Holmes, 1983; Kuramoto, 2003). Theoretically, oscillators are classified according to their bifurcation types, such as saddle-node and Hopf bifurcations. Oscillation by the saddle-node bifurcation emerges when a half-stable cycle splits into a pair of stable and unstable limit cycles, but oscillation by the Hopf bifurcation emerges when a stable spiral fixed point changes to a unstable spiral fixed point surrounded by a stable limit cycle (Hale and Koçak, 1991; Strogatz, 2001). Although phase space structures can be partly derived from such bifurcation types, the dynamic properties of the system have to be evaluated by other methods. To investigate the underlying oscillatory mechanism, a framework termed the phase reduction method has been developed in mathematics and non-linear physics (Hansel et al., 1995; Hoppensteadt and Izhikevich, 1997; Kuramoto, 2003). By using this method, an oscillator in a high dimensional space can be described by only one variable, phase, and its dynamics are packed into a phase response (or phase sensitivity) function. The phase response function has been derived analytically, not only from mathematical models but also from experimental biological data (Reyes and Fetz, 1993; Khalsa et al., 2003; Lahav et al., 2004; Stricker et al., 2008). This method facilitates the classification of structurally related but dynamically differentiated biochemical oscillators. The phase response functions have been classified into two types, commonly referred to as Type I and Type II (Hansel et al., 1995; Hoppensteadt and Izhikevich, 1997; Kuramoto, 2003). A Type I phase response function generally attains a positive value during an oscillation period, whereas a Type II phase response function possesses a significantly large region of negative values. A small perturbation to an oscillator advances its phase when it is in a phase that generates a positive phase response, but retards its phase when in a negative phase response. It is known that Type I and Type II phase response functions correspond to saddle-node and Hopf bifurcations, respectively (Rinzel and Ermentrout, 1998).

In this study we have used three models and the phase reduction method to investigate the type of phase response function and phase difference of two weakly coupled oscillators in the steady state.

## 2 Results

### 2.1 Single oscillator: A phosphorylation-dephosphorylation cycle

**Figure 1:**
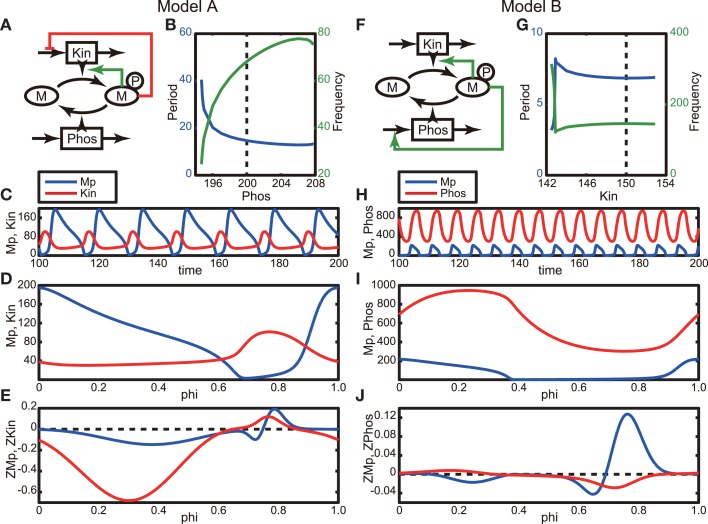
**Phosphorylation-dephosphorylation cycle models**. **(A)** Schematic of Model A, in which the phosphorylated M, Mp, decreases the production of kinase or inhibits its activity. Here, Phos takes a constant value, 200. **(B)** Period (blue line) and frequency (green line) of oscillation as a function of Phos. **(C)** Oscillatory behavior of the model network. Blue and red lines correspond to Mp and Kin, respectively. **(D)** Periodic orbit during one period of the model. The horizontal axis represents the phase. Colors are the same as in **(C)**. **(E)** Phase response functions of the model, which are obtained by solving the adjoint equation given in Equation 4 (Ermentrout, 1996). Colors are the same as in **(C)**. **(F)** Schematic of Model B, in which the phosphorylated M, Mp, increases the production of phosphatase or activates its activity. Here, Kin takes a constant value, 150. **(G–J)** Same as in **(B–E)**, but here they are obtained using model B. Blue and red lines in **(H–J)** correspond to Mp and Phos, respectively.

Several modes of negative feedback regulation have been identified in signal transduction pathways, and these are potential candidates for emerging oscillatory phenomena. Many fundamental negative feedback models that cause the emergence of oscillations have been proposed (Kholodenko, 2006; Novák and Tyson, 2008). Here, we adopt the simple phosphorylation-dephosphorylation cycle models proposed by Kholodenko (2006). While he has considered all the possible topologies of feedback regulation to phosphorylation and dephosphorylation steps in the cycle, we use two, Models A and B (the latter of which corresponds to Model C in the original paper) in our study (Figures 1A,F, Section 4.2). In these models, negative feedback is realized by inhibiting kinase (Kin) production (or its activity) in Model A and enhancing phosphatase (Phos) production (or its activity) in Model B. First, we explore the parameters exhibiting oscillation by varying Phos and Kin for Models A and B, respectively (Figure 1). The parameter regions that can induce oscillations, resultant oscillation periods, and frequencies are shown in Figures 1B,G. The long-dashed lines indicate the parameter values that we have adopted in the following analyses. Figures 1C,H show the periodic orbits of the models. The oscillation periods in the two models are clearly very different from each other.

We next applied a phase reduction method to these models and calculated the phase response functions of Models A and B. The details of phase reduction method are found in Section 4.1, in which the state vector ***X***(***t***) is given by (Mp(*t*), Kin(*t*)) and (Mp(*t*), Phos(*t*)), where Mp represents an activated and phosphorylated form of M, for Models A and B, respectively. Figures 1D,I show the periodic orbits for one oscillation period in Models A and B, respectively, in which the peaks of the Mp (blue lines) are located at the origin of the phase. The phase response functions of Mp of both models are similar to each other and have significantly large regions of both positive and negative values (Figures 1E,J for Models A and B, respectively), which means that they can be categorized as Type II oscillators. This result implies that these regulatory networks have similar phase responses of Mp to a small perturbation regardless of the difference in their biological feedback targets.

### 2.2 Coupled oscillators: Interacting phosphorylation-dephosphorylation cycles

**Figure 2:**
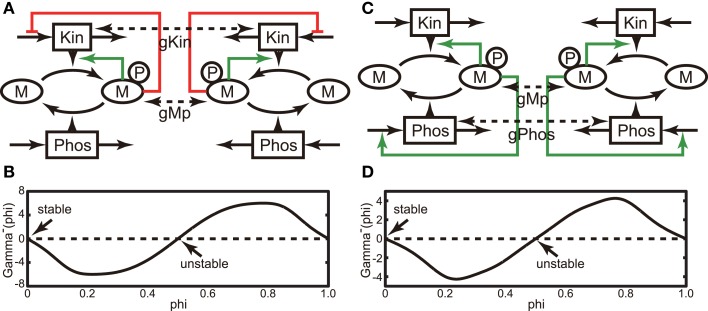
**(A)** Schematic of a pair of the identical oscillators of Model A. Here, we assume weak interactions between each Mp and Kin. **(B)** Gamma function of the coupled system shown in **(A)**, in which we adopt *g*Mp = 1 and *g*Kin = 1. **(C)** Same as **(A)**, but Model B was used by assuming weak interactions between each Mp and Kin. **(D)** Gamma function of the coupled system shown in **(C)**, in which we adopted *g*Mp = 1 and *g*Phos = 1.

Next, we investigated the behavior of the above models in the presence of a weak interaction in the steady state by presuming that two cells are located next to each other. The phase reduction method allows calculation of the fixed phase difference of a weakly coupled pair of identical oscillators in the steady state (for details, see Section 4.1). Here, we consider the case of two identical oscillators interacting through a weak diffusion coupling (see Figures 2A,C). In addition to the model equations for Models A and B (Section 4.2), we adopt the following diffusion couplings. For Model A, the interaction function in Equations (7) and (8) is given by ***C***((Mp*_i_*(*t*), Kin*_i_*(*t*)), (Mp*_j_*(*t*), Kin*_j_*(*t*))) = (−*g*Mp(Mp*_i_*(*t*) − Mp*_j_*(*t*)), −*g*Kin(Kin*_i_*(*t*) − Kin*_j_*(*t*))). Similarly, for Model B, it is given by ***C***((Mp*_i_*(*t*), Phos*_i_*(*t*)), (Mp*_j_*(*t*), Phos*_j_*(*t*))) = (−*g*Mp(Mp*_i_*(*t*) − Mp*_j_*(*t*)), −*g*Phos(Phos*_i_*(*t*) − Phos*_j_*(*t*))). We assume that the coefficients of interactions, *g*Mp, *g*Kin, and *g*Phos, are sufficiently small for each oscillator to remain in the basin of the periodic orbit. Under weak coupling conditions, a coupled dynamical system can be reduced to a system governed only by phase difference. The reduced dynamical system is given by Equations (17) and (18). Once we obtain the phase response function, we can calculate the gamma function (Γ ^−^(*Φ*), defined by Equations (17) and (18)), thereby delineating the dynamics of the phase difference. The gamma functions for Models A and B are shown in Figures 2B,D, respectively. As shown in the figures, *Φ* = 0 and 0.5 satisfy Γ ^−^(*Φ*) = 0 for both models, and only *Φ* = 0 satisfies the stability conditions (Equations (19) and (20)). Therefore, the oscillators in both coupled dynamical systems are asymptotically synchronized in the steady state. Next, we evaluated the shape of the gamma function by varying the ratio *g*Kin*/g*Mp (*g*Phos*/g*Mp) between 0 and 1 for Model A (Model B) and find that *Φ* = 0 is the only stable solution in this parameter region. Therefore, these results suggest that in a many body system consisting of each model, their oscillation cycles can synchronize with an almost zero phase difference in a noisy environment.

### 2.3 MAPK cascade model

**Figure 3:**
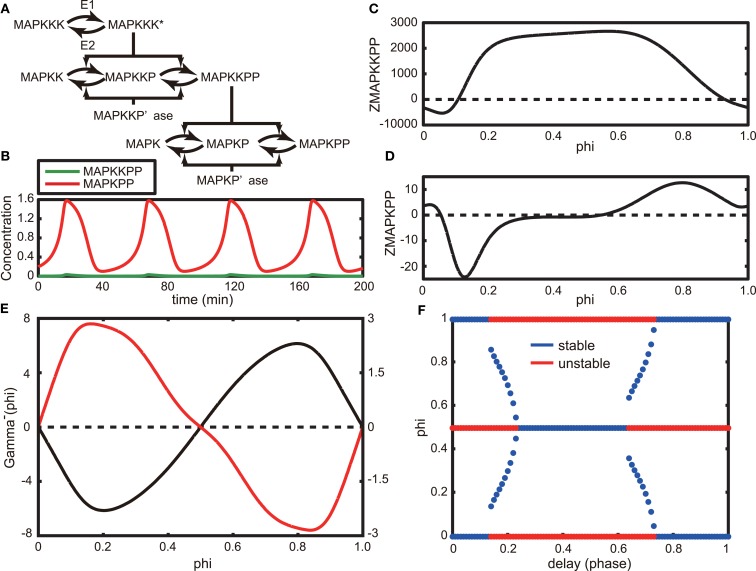
**(A)** Schematic of the Huang-Ferrell model. Here, MAPKKK* indicates the activation form of MAPKKK. **(B)** Oscillatory behaviors of MAPKPP and MAPKPP in the steady state of the model. **(C,D)** Phase response functions of MAPKPP **(C)** and MAPKPP **(D)**, respectively. **(E)** Gamma function without a time delay (black line) and one with a time delay *τ* = 0.25 (red line). **(F)** Stable (blue) and unstable (red) fixed phase differences as a function of time delay *τ*.

Next, we considered the asynchronous oscillations experimentally observed by Shankaran et al. (2009). They reported that EGF induces oscillations in the nuclear localization (an indication of activation) of ERK in living cells and that the oscillations are asynchronous between neighboring cells (Figure 1 in Shankaran et al., 2009). Here, we adopt the MAPK cascade model that was originally developed by Huang and Ferrell (1996) and later modified by Qiao et al. (2007). The Huang-Ferrell model has been widely used for analyzing the dynamic behavior of the MAPK system (for example, Ferrell and Machleder, 1998). The original model and parameters produce an ultrasensitive MAPK activity with a Hill coefficient of 4-5, but later, Qiao et al. found that the model can also produce oscillations with other parameter sets in a wide range of the parameter space. Here, we used the Huang-Ferrell model modified by Qiao et al. to analyze the oscillatory behavior of MAPK activity (Figure 3A, Section 4.3).

The time evolutions of MAPKKPP and MAPKPP in an oscillatory state are shown in Figure 3B. Here, we assume that MAPKPP is an activated ERK as observed by Shankaran et al. We applied the phase reduction method to this model and evaluated the stable fixed phase difference by calculating the phase response function of the model. The phase response functions of MAPKKPP and MAPKPP are shown in Figures 3C,D, respectively, in which the origin of the phase corresponds to the peak of MAPKPP. As shown in the Figure 3D, MAPKPP showed a Type II phase response, the same as those of Models A and B described in the previous section.

Next, we considered the phase difference of a pair of identical MAPK oscillators. Because the entire signal transduction network in the cells used by Shankaran et al. remains unknown, we substituted a MAPK cascade as an EGF-induced signaling pathway in the observed neighboring cells. In addition, we assumed that the interaction between a pair of cells can be effectively modeled as a function of the difference between the MAPKPPs. Then we adopted a simple diffusion type interaction function: – *g*MAPKPP(MAPKPP*_i_*(*t*) – MAPKPP*_j_*(*t*)). Using this function, we calculated the gamma function as shown in Figure 3E (black line). The value *Φ* = 0 is the only stable solution and the two MAPK oscillators are synchronized asymptotically. Thus, employment of only a diffusion interaction between the two MAPK models (or cells) was insufficient to reproduce the asynchronous oscillation of MAPKPP that has been detected in living cells.

To achieve an asynchronous oscillation, the sign of the coefficient can be changed. When doing so, we obtained a gamma function whose shape is the reflection of the one shown as a black line in Figure 3E and *Φ* = 0.5 results in a stable solution. Because we considered an effective interaction in our model, a negative diffusion coefficient can be considered. Another possible scenario is to incorporate a time delay within the interaction, – *g*MAPKPP(MAPKPP*_i_*(*t*) – MAPKPP*_j_*(*t* − *τ*)), where *τ* > 0. This scenario is reasonable because spatially isolated cells need to communicate with each other, but the signal from one cell will be delivered with a time delay to another cell when the two cells remain apart. In agreement with this theoretical assumption, oscillatory ERK activity was only observed in cells grown at low density but not in cells at confluency (Shankaran et al., 2009). When we incorporated a time delay into the interaction, the gamma function could be very simply calculated (Equations (30) and (31)). Considering *τ* = 0.25 as a delay time, we calculated the gamma function in Equation (31), as illustrated in Figure 3E (red line). As a result, *Φ* = 0.5 transforms into a stable solution. Therefore, ERK signals potentially asynchronously oscillate in the delayed system. In Figure 3F, we show how stable and unstable fixed phase solutions alter by varying the time delay, *τ*, between 0 and 1. A time delay 0.24 ≤ *τ* ≤ 0.63 results in *Φ* = 0.5, which is the only stable fixed phase difference. Interestingly, a wide range of time delays could induce asynchronous oscillations of MAPK.

## 3 Discussion

In this study, we first evaluated the phase response function, a characteristic property related to oscillation, for two simple phosphorylation-dephosphorylation cycle models A and B, in which negative feedback regulation either inhibits the kinase activity or enhances the phosphatase activity. The two models have relatively different oscillatory periods. However, their phase response functions corresponding to the reaction product Mp are very similar and both have a Type II phase response, i.e., their phase can be retarded or advanced depending on the timing of an external stimulus. This result suggests that even if cells use different negative feedback regulatory mechanisms (e.g., kinase inhibition or phosphatase activation), they can produce similar, although not exactly the same, oscillatory dynamics. It also indicates that similar oscillatory signals can be generated using different biological components and resources, such as kinases and phosphatases, produced by regulated transcription or activated by post-transcriptional modification in a cell context manner. Furthermore, we investigated the fixed phase differences of a pair of identical oscillators interacting through weak diffusion coupling. Here we found that the oscillations originating from each model can synchronize even if their interaction is weak. This result implies that when two almost identical oscillations (i.e., the biochemical reaction dynamics generated from Model A or B) can interact in spatially separated subcellular locations (such as the cytoplasm and nucleus), their oscillatory signals can still be synchronized. These results suggest that oscillation dynamics can be robustly maintained within the cell.

We next analyzed the MAPK cascade and found that the phase response function corresponding to MAPKPP is also Type II. When two identical MAPK oscillators are coupled through diffusion, these signals are synchronized. Again, in addition to the above two models, this result confirmed that oscillatory signals can be robust within the cell. However, the presumed interaction of MAPKs mediated by diffusion in our analysis did not satisfactorily reproduce the asynchronous ERK oscillation dynamics reported by Shankaran et al. (2009). We reasoned that this inconsistency could be the result of a time delay in the coupling and, under this condition, asynchronous oscillation was observed with a wide range of parameters in time delays. Our results suggest that whether a pair of cells oscillates synchronously or asynchronously may depend on the distance between the cells. In the experimental setting of Shankaran et al., oscillatory ERK activity was only observed in cells grown at low density but not in confluent cells. Therefore, we presume that compensatory mechanisms would mask the oscillatory dynamics of ERK in cells at high density. In reality, thousands of MAPK molecules exist in a cell, therefore molecular regulation of MAPK in a cell in a tightly packed cell population may be quite different from that in a sparse cell population, and therefore the former circumstance would interfere with experimental visualization of oscillatory dynamics of MAPK.

In this study, we only dealt with identical pathways under weak interaction to examine the effect of two units coupling for an oscillatory response. However, our current study suggests that a pair of slightly different oscillators under weak interaction conditions can result in similar synchronous behavior: therefore the method should be applicable for an evaluation of non-identical oscillatory units as well. The phase reduction method thus can be applied to pairs of widely different oscillator modules, as are frequently observed in biological networks.

## 4 Materials and Methods

### 4.1 Phase reduction method

The phase reduction method has been widely applied to coupled oscillator systems. Here, we briefly describe the derivation of the phase model. First, we define the phase. We consider a system whose dynamics is described in the vector form as follows:

(1) $$\frac{d\text{X}\left(t\right)}{dt}=\text{F}\left(\text{X}\left(t\right)\right),$$

where ***X***(*t*) represents the dynamical variable, and ***F***(***X***(*t*)) is a vector function that determines the dynamics of the system. Hereafter, we restrict the phase space of the system to a region in its basin of attraction in which a stable limit cycle, χ(*t*), exists. We assume that the period of the solution is *T*, i.e., χ(*t* + *T*) = χ(*t*). According to the phase reduction method, such a system can be reduced to a system consisting of the phase degree of freedom only. We can define a phase Φ(*t*) ∈ [0, 1) along the periodic orbit χ(*t*) with constant time derivative as follows:

(2) $$\frac{d\phi \left(t\right)}{dt}=1,$$

in which we can define the origin arbitrarily. Because the periodic orbit can be parameterized by *Φ*(*t*), we describe it by χ(*Φ*(*t*)). Although the phase is defined along the periodic orbit, it can be extended into the neighborhood of the periodic orbit, which is known as the asymptotic phase. Because the asymptotic phase can be described as a function of the state ***X***(*t*), we can derive its dynamical equation as follows:

(3) $$\begin{array}{ll} \frac{d\phi \left(\text{X}\left(t\right)\right)}{dt} & =\frac{\partial \phi \left(\text{X}\right)}{\partial X}\cdot \frac{d\text{X}\left(t\right)}{dt}\\ & =\frac{\partial \phi \left(\text{X}\right)}{\partial \text{X}}\cdot \text{F}\left(\text{X}\left(t\right)\right), \end{array}$$

where · denotes the inner product. From Equations (2) and (3), *Φ*(***X***(*t*)) must satisfy the following expression

(4) $$\frac{\partial \phi \left(\text{X}\right)}{\partial \text{X}}\cdot \text{F}\left(\text{X}\right)=1.$$

Next, we derive the phase response of the system. We assume that the system Equation (1) receives a weak perturbation:

(5) $$\frac{d\text{X}\left(t\right)}{dt}=\text{F}\left(\text{X}\left(t\right)\right)+\text{p}\left(t\right).$$

Similar to Equation (3), the asymptotic phase obeys the following dynamical equation:

(6) $$\begin{array}{ll} \frac{d\phi \left(\text{X}\left(t\right)\right)}{dt} & =\frac{\partial \phi \left(\text{X}\right)}{\partial \text{X}}\cdot \frac{d\text{X}\left(t\right)}{dt}\\ & =\frac{\partial \phi \left(\text{X}\right)}{\partial \text{X}}\cdot \left(\text{F}\left(\text{X}\left(t\right)\right)+\text{p}\left(t\right)\right)\\ & =1+\frac{\partial \phi \left(\text{X}\right)}{\partial \text{X}}\cdot \text{p}\left(t\right)\\ & =1+\text{Z}\left(\phi \right)\cdot \text{p}\left(t\right), \end{array}$$

in which ***Z***(*Φ*) is called as the “phase response function” or “phase sensitivity.” The phase response function describes the phase response (phase shift) of the system to small perturbations.

Now, we consider a coupled oscillator system consisting of two identical oscillatory units,

(7) $$\begin{array}{lll} & \frac{d{\text{X}}_{1}\left(t\right)}{dt}=\text{F}\left({\text{X}}_{1}\left(t\right)\right)+\text{C}\left({\text{X}}_{1}\left(t\right),{\text{X}}_{2}\left(t\right)\right), & \end{array}$$

(8) $$\begin{array}{lll} & \frac{d{\text{X}}_{2}\left(t\right)}{dt}=\text{F}\left({\text{X}}_{2}\left(t\right)\right)+\text{C}\left({\text{X}}_{2}\left(t\right),{\text{X}}_{1}\left(t\right)\right). & \end{array}$$

Here, ***X****_i_*(*t*) represents the dynamical variables corresponding to the *i*-th unit, and ***C*** is the interaction function. When the interaction is weak (“weak” means that the units do not leave the basin of attraction of the periodic orbit), the coupled system can be described by the asymptotic phase as follows:

(9) $$\begin{array}{lll} \frac{d\phi_{1}\left(t\right)}{dt} & =1+\text{Z}\left(\phi_{1}\left(t\right)\right)\cdot \text{C}\left(\phi_{1}\left(t\right),\phi_{2}\left(t\right)\right), & \end{array}$$

(10) $$\begin{array}{lll} \frac{d\phi_{2}\left(t\right)}{dt} & =1+\text{Z}\left(\phi_{2}\left(t\right)\right)\cdot \text{C}\left(\phi_{2}\left(t\right),\phi_{1}\left(t\right)\right). & \end{array}$$

By replacing the phase *Φ*_1,2_(*t*) with the phase ψ_1,2_(*t*) defined by *Φ*_1,2_(*t*) = *t* + ψ_1,2_(*t*), Equations (9) and (10) are transformed into the following equations:

(11) $$\begin{array}{lll} & \frac{d\psi_{1}\left(t\right)}{dt}=\Gamma \left(\psi_{1}\left(t\right)-\psi_{2}\left(t\right)\right), & \end{array}$$

(12) $$\begin{array}{lll} & \frac{d\psi_{2}\left(t\right)}{dt}=\Gamma \left(\psi_{2}\left(t\right)-\psi_{1}\left(t\right)\right), & \end{array}$$

where

(13) $$\Gamma \left(\psi_{i}\left(t\right)-\psi_{j}\left(t\right)\right)=\int_{0}^{1}d\theta \text{Z}\left(\theta +\psi_{i}\left(t\right)\right)\cdot \text{C}\left(\theta +\psi_{i}\left(t\right),\theta +\psi_{j}\left(t\right)\right),$$

in which we used the fact that ψ_1,2_(*t*) vary slowly, the weak interaction assumption, and the average during one period. By substituting ψ_1,2_(*t*) = −*t* + *Φ*_1,2_(*t*) into Equations (11–13), we obtain the phase description of the coupled oscillator system as follows:

(14) $$\begin{array}{lll} \frac{d\phi_{1}\left(t\right)}{dt} & =1+\Gamma \left(\phi_{1}\left(t\right),\phi_{2}\left(t\right)\right), & \end{array}$$

(15) $$\begin{array}{lll} \frac{d\phi_{2}\left(t\right)}{dt} & =1+\Gamma \left(\phi_{2}\left(t\right),\phi_{1}\left(t\right)\right), & \end{array}$$

(16) $$\begin{array}{lll} \Gamma \left(\phi_{i}\left(t\right),\phi_{j}\left(t\right)\right) & =\int_{0}^{1}d\theta \text{Z}\left(\theta +\phi_{i}\left(t\right)\right)\cdot \text{C}\left(\theta +\phi_{i}\left(t\right),\theta +\phi_{j}\left(t\right)\right). & \end{array}$$

By subtracting Equation (15) from Equation (14), the ordinary differential equation (ODE) is obtained that governs the evolution of the phase difference *Φ*(*t*) = *Φ*_1_(*t*) − *Φ*_2_(*t*),

(17) $$\begin{array}{lll} \frac{d\phi \left(t\right)}{dt} & =\Gamma^{-}\left(\phi \left(t\right)\right)\equiv \Gamma \left(\phi \left(t\right)\right)-\Gamma \left(-\phi \left(t\right)\right), & \end{array}$$

(18) $$\begin{array}{lll} \Gamma \left(\phi \left(t\right)\right) & =\int_{0}^{1}d\theta \text{Z}\left(\theta +\phi \left(t\right)\right)\cdot \text{C}\left(\theta +\phi \left(t\right),\theta \right). & \end{array}$$

The fixed phase difference *Φ* is stable if it satisfies the following conditions:

(19) $$\begin{array}{lll} \Gamma^{-}\left(\phi \right)\; & =\;0, & \end{array}$$

(20) $$\begin{array}{lll} \frac{d\Gamma^{-}\left(\phi \right)}{d\phi } & <0, & \end{array}$$

and it is unstable if it satisfies the following conditions:

(21) $$\begin{array}{lll} \Gamma^{-}\left(\phi \right) & =0, & \end{array}$$

(22) $$\begin{array}{lll} \frac{d\Gamma^{-}\left(\phi \right)}{d\phi } & >0. & \end{array}$$

Finally, we derive a phase model corresponding to a coupled oscillator system with a delayed interaction. In this case, the system is expressed as follows:

(23) $$\begin{array}{lll} & \frac{d{\text{X}}_{1}\left(t\right)}{dt}=\text{F}\left({\text{X}}_{1}\left(t\right)\right)+\text{C}\left({\text{X}}_{1}\left(t\right),{\text{X}}_{2}\left(t-\tau \right)\right), & \end{array}$$

(24) $$\begin{array}{lll} & \frac{d{\text{X}}_{2}\left(t\right)}{dt}=\text{F}\left({\text{X}}_{2}\left(t\right)\right)+\text{C}\left({\text{X}}_{2}\left(t\right),{\text{X}}_{1}\left(t-\tau \right)\right). & \end{array}$$

Then, Equations (9) and (10) are changed into the following equations:

(25) $$\begin{array}{lll} & \frac{d\phi_{1}\left(t\right)}{dt}=1+\text{Z}\left(\phi_{1}\left(t\right)\right)\cdot \text{C}\left(\phi_{1}\left(t\right),\phi_{2}\left(t-\tau \right)\right), & \end{array}$$

(26) $$\begin{array}{lll} & \frac{d\phi_{2}\left(t\right)}{dt}=1+\text{Z}\left(\phi_{2}\left(t\right)\right)\cdot \text{C}\left(\phi_{2}\left(t\right),\phi_{1}\left(t-\tau \right)\right). & \end{array}$$

In a first order approximation, we can assume that *Φ*_1,2_(*t* − *τ*) = *Φ*_1,2_(*t*) − *τ*. Then, the phase description of the system is given by the following equations:

(27) $$\begin{array}{lll} \frac{d\phi_{1}\left(t\right)}{dt} & =1+\Gamma \left(\phi_{1}\left(t\right),\phi_{2}\left(t\right)\right), & \end{array}$$

(28) $$\begin{array}{lll} \frac{d\phi_{2}\left(t\right)}{dt} & =1+\Gamma \left(\phi_{2}\left(t\right),\phi_{1}\left(t\right)\right), & \end{array}$$

(29) $$\begin{array}{llll} \Gamma \left(\phi_{i}\left(t\right),\phi_{j}\left(t\right)\right) & =\int_{0}^{1}d\theta \text{Z}\left(\theta +\phi_{i}\left(t\right)\right)\cdot \text{C}(\theta +\phi_{i}\left(t\right),\theta & & \\ & \;+\phi_{j}\left(t\right)-\tau ). & \end{array}$$

Therefore, the ODE of the phase difference attains the following form:

(30) $$\begin{array}{lll} \frac{d\phi \left(t\right)}{dt} & =\Gamma^{-}\left(\phi \left(t\right)\right)\equiv \Gamma \left(\phi \left(t\right)\right)-\Gamma \left(-\phi \left(t\right)\right), & \end{array}$$

(31) $$\begin{array}{lll} \Gamma \left(\phi \left(t\right)\right) & =\int_{0}^{1}d\theta \text{Z}\left(\theta +\phi \left(t\right)\right)\cdot \text{C}\left(\theta +\phi \left(t\right),\theta -\tau \right), & \end{array}$$

in which the delay is no longer explicitly included in the equation.

### 4.2 Simple phosphorylation model

Model A

(32) $$\begin{array}{lll} \frac{dMp}{dt} & =v_{kin}-v_{phos} & \end{array}$$

(33) $$\begin{array}{lll} & =\frac{k_{kin}^{cat}KinM}{K_{m1}+M}\cdot \frac{1+AMp∕K_{a}}{1+Mp∕K_{a}}-\frac{k_{phos}^{cat}PhosMp}{K_{m2}+Mp}, & \end{array}$$

(34) $$\begin{array}{lll} \frac{dKin}{dt} & =v_{kin}^{synth}-v_{kin}^{deg} & \end{array}$$

(35) $$\begin{array}{lll} & =V_{kin}^{0}\frac{1+Mp∕K_{I}}{1+I\cdot Mp∕K_{I}}-k_{kin}^{deg}Kin, & \end{array}$$

(36) $$\begin{array}{lll} M & =M^{tot}-Mp. & \end{array}$$

Model B

(37) $$\begin{array}{lll} \frac{dMp}{dt} & =v_{kin}-v_{phos} & \end{array}$$

(38) $$\begin{array}{lll} & =\frac{k_{kin}^{cat}KinM}{K_{m1}+M}\cdot \frac{1+AMp∕K_{a}}{1+Mp∕K_{a}}-\frac{k_{phos}^{cat}PhosMp}{K_{m2}+Mp}, & \end{array}$$

(39) $$\begin{array}{lll} \frac{dPhos}{dt} & =v_{phos}^{synth}-v_{phos}^{deg} & \end{array}$$

(40) $$\begin{array}{lll} & =V_{phos}^{0}\frac{1+A_{dp}Mp∕K_{d}}{1+Mp∕K_{d}}-k_{phos}^{deg}Phos, & \end{array}$$

(41) $$\begin{array}{lll} M & =M^{tot}-Mp. & \end{array}$$

The parameters of Models A and B are given below.

**Table:** 

Parameter	Model A	Model B
*M^tot^*	300.0	300.0
$k_{kin}^{cat}$	1.0	1.0
*A*	100.0	100.0
*K_a_*	500.0	500.0
*K*_*m*1_	500.0	500.0
$k_{phos}^{cat}$	1.0	1.0
*K*_*m*2_	10.0	10.0
*Phos*(A), *Kin*(B)	200.0	150.0
$v_{kin}^{0}\left(\text{A}\right),V_{phos}^{0}\left(\text{B}\right)$	150.0	200.0
*K_I_*(A), *K_d_*(B)	100.0	100.0
*I*(A), *A_dp_*(B)	7.5	7.5
$k_{kin}^{deg}\left(\text{A}\right),k_{phos}^{deg}\left(B\right)$	1.0	1.0

### 4.3 Huang-Ferrell model

The ODEs of the model are as follows:

(42) $$\begin{array}{lll} \frac{dv_{0}}{dt} & =a_{0}c_{0}c_{1}-\left(d_{0}+k_{0}\right)v_{0}, & \end{array}$$

(43) $$\begin{array}{llll} \frac{dv_{1}}{dt} & =k_{0}v_{0}-a_{1}v_{1}c_{2}+d_{1}v_{2}-a_{2}v_{1}c_{3}+\left(k_{2}+d_{2}\right)v_{3} & & \\ & \;-a_{4}v_{4}v_{1}+\left(k_{4}+d_{4}\right)v_{6}, & \end{array}$$

(44) $$\begin{array}{lll} \frac{dv_{2}}{dt} & =a_{1}v_{1}c_{2}-\left(d_{1}+k_{1}\right)v_{2}, & \end{array}$$

(45) $$\begin{array}{lll} \frac{dv_{3}}{dt} & =a_{2}c_{3}v_{1}-\left(d_{2}+k_{2}\right)v_{3}, & \end{array}$$

(46) $$\begin{array}{lll} \frac{dv_{4}}{dt} & =k_{2}v_{3}-a_{3}v_{4}c_{4}+d_{3}v_{5}-a_{4}v_{4}v_{1}+d_{4}v_{6}+k_{5}v_{8}, & \end{array}$$

(47) $$\begin{array}{lll} \frac{dv_{5}}{dt} & =a_{3}v_{4}c_{4}-\left(d_{3}+k_{3}\right)v_{5}, & \end{array}$$

(48) $$\begin{array}{lll} \frac{dv_{6}}{dt} & =a_{4}v_{4}v_{1}-\left(d_{4}+k_{4}\right)v_{6}, & \end{array}$$

(49) $$\begin{array}{llll} \frac{dv_{7}}{dt} & =k_{4}v_{6}-a_{5}v_{7}c_{4}+d_{5}v_{8}-a_{6}v_{7}c_{5}+\left(d_{6}+k_{6}\right)v_{9} & & \\ & \;-a_{8}v_{10}v_{7}+\left(d_{8}+k_{8}\right)v_{12}, & \end{array}$$

(50) $$\begin{array}{lll} \frac{dv_{8}}{dt} & =a_{5}v_{7}c_{4}-\left(d_{5}+k_{5}\right)v_{8}, & \end{array}$$

(51) $$\begin{array}{lll} \frac{dv_{9}}{dt} & =a_{6}c_{5}v_{7}-\left(d_{6}+k_{6}\right)v_{9}, & \end{array}$$

(52) $$\begin{array}{lll} \frac{dv_{10}}{dt} & =k_{6}v_{9}-a_{7}v_{10}c_{6}+d_{7}v_{11}-a_{8}v_{10}v_{7}+d_{8}v_{12}+k_{9}v_{14}, & \end{array}$$

(53) $$\begin{array}{lll} \frac{dv_{11}}{dt} & =a_{7}v_{10}c_{6}-\left(d_{7}+k_{7}\right)v_{11}, & \end{array}$$

(54) $$\begin{array}{lll} \frac{dv_{12}}{dt} & =a_{8}v_{10}v_{7}-\left(d_{8}+k_{8}\right)v_{12}, & \end{array}$$

(55) $$\begin{array}{lll} \frac{dv_{13}}{dt} & =k_{8}v_{12}-a_{9}v_{13}c_{6}+d_{9}v_{14}, & \end{array}$$

(56) $$\begin{array}{lll} \frac{dv_{14}}{dt} & =a_{9}v_{13}c_{6}-\left(d_{9}+k_{9}\right)v_{14}. & \end{array}$$

The algebraic equations and total quantities of the molecules are as follows:

(57) $$\begin{array}{lll} c_{0} & =\text{KK}{\text{K}}_{\text{tot}}-\left(v_{0}+v_{1}+v_{2}+v_{3}+v_{6}\right), & \end{array}$$

(58) $$\begin{array}{lll} c_{1} & =\text{E}{\text{1}}_{\text{tot}}-v_{0}, & \end{array}$$

(59) $$\begin{array}{lll} c_{2} & =\text{E}{\text{2}}_{\text{tot}}-v_{5}, & \end{array}$$

(60) $$\begin{array}{llll} c_{3} & =\text{K}{\text{K}}_{\text{tot}}-(v_{3}+v_{4}+v_{5}+v_{6}+v_{7} & & \\ & \;+v_{8}+v_{9}+v_{12}), & \end{array}$$

(61) $$\begin{array}{lll} c_{4} & ={\text{KKP}}^{\prime }\text{as}{\text{e}}_{\text{tot}}-\left(v_{5}+v_{8}\right), & \end{array}$$

(62) $$\begin{array}{lll} c_{5} & ={\text{K}}_{\text{tot}}-\left(v_{9}+v_{10}+v_{11}+v_{12}+v_{13}+v_{14}\right), & \end{array}$$

(63) $$\begin{array}{lll} c_{6} & ={\text{KP}}^{\prime }\text{as}{\text{e}}_{\text{tot}}-\left(v_{11}+v_{14}\right), & \end{array}$$

(64) $$\begin{array}{lll} \text{KK}{\text{K}}_{\text{tot}} & =3.7112E-003, & \end{array}$$

(65) $$\begin{array}{lll} \text{E}{\text{1}}_{\text{tot}} & =1.0000E-007, & \end{array}$$

(66) $$\begin{array}{lll} \text{E}{\text{2}}_{\text{tot}} & =1.2115E-004, & \end{array}$$

(67) $$\begin{array}{lll} \text{K}{\text{K}}_{\text{tot}} & =4.0658, & \end{array}$$

(68) $$\begin{array}{lll} {\text{KKP}}^{\prime }\text{as}{\text{e}}_{\text{tot}} & =9.4579E-005, & \end{array}$$

(69) $$\begin{array}{lll} {\text{K}}_{\text{tot}} & =2.0, & \end{array}$$

(70) $$\begin{array}{lll} {\text{KP}}^{\prime }\text{as}{\text{e}}_{\text{tot}} & =7.5372E-002. & \end{array}$$

The kinetic parameters are given below:

**Table:** 

*a*_0_ = 1.0962*E* + 003	*d*_0_ = 4.3207*E* + 001	*k*_0_ = 6.9525*E* + 001
*a*_1_ = 1.5576*E* + 003	*d*_1_ = 9.4394*E* + 001	*k*_1_ = 2.8874*E* + 002
*a*_2_ = 1.9179*E* + 003	*d*_2_ = 7.5216*E* + 001	*k*_2_ = 4.3432*E* + 001
*a*_3_ = 3.6894*E* + 002	*d*_3_ = 4.1710*E* + 002	*k*_3_ = 7.1505*E* + 002
*a*_4_ = 4.5836*E* + 003	*d*_4_ = 3.3742*E* + 002	*k*_4_ = 1.6957*E* + 002
*a*_5_ = 2.0219*E* + 003	*d*_5_ = 4.3905*E* + 002	*k*_5_ = 3.4842*E* + 002
*a*_6_ = 2.6634*E* + 003	*d*_6_ = 5.9598*E* + 001	*k*_6_ = 4.1954*E* + 001
*a*_7_ = 1.4435*E* + 003	*d*_7_ = 4.0101*E* + 001	*k*_7_ = 6.3433*E* + 001
*a*_8_ = 5.4366*E* + 002	*d*_8_ = 2.2211*E* + 002	*k*_8_ = 1.1716*E* + 002
*a*_9_ = 4.3990*E* + 002	*d*_9_ = 1.7642*E* + 002	*k*_9_ = 4.6705*E* + 001

Molecular names, variables, and initial conditions.

**Table:** 

Molecular name	Variable	Initial condition
*KKK* · *E*_1_	*v*_0_	3.3875*E* − 009
KKK*	*v*_1_	1.6561*E* − 006
KKK* · E_2_	*v*_2_	8.1569*E* − 010
KK · KKK*	*v*_3_	1.0056*E* − 004
KKP	*v*_4_	2.1568*E* − 001
KKP · KKP′ase	*v*_5_	6.1078*E* − 006
KKP · KKK*	*v*_6_	3.2293*E* − 006
KKPP	*v*_7_	7.0439*E* − 003
KKPP · KKP′ase	*v*_8_	1.5716*E* − 006
*K* · *KKPP*	*v*_9_	8.1041*E* − 002
KP	*v*_10_	4.9431*E* − 001
KP · KP′ase	*v*_11_	5.3600*E* − 002
KP · KKPP	*v*_12_	5.5795*E* − 003
KPP	*v*_13_	9.1279*E* − 001
KPP · KP′ase	*v*_14_	1.3996*E* − 002

## Conflict of Interest Statement

The authors declare that the research was conducted in the absence of any commercial or financial relationships that could be construed as a potential conflict of interest.

## Research Topic:

From structural to molecular systems biology: experimental and computational approaches to unravel mechanisms of kinase activity regulation in cancer and neurodegeneration

## Topic Editor:

Matteo Barberis, Humboldt University Berlin, Germany; Max Planck Institute for Molecular Genetics, Berlin, Germany
